# Locomotion Control With Frequency and Motor Pattern Adaptations

**DOI:** 10.3389/fncir.2021.743888

**Published:** 2021-11-25

**Authors:** Mathias Thor, Beck Strohmer, Poramate Manoonpong

**Affiliations:** ^1^Embodied AI and Neurorobotics Lab, SDU Biorobotics, The Mærsk Mc-Kinney Møller Institute, The University of Southern Denmark, Odense, Denmark; ^2^Bio-Inspired Robotics and Neural Engineering Laboratory, School of Information Science and Technology, Vidyasirimedhi Institute of Science and Technology, Rayong, Thailand

**Keywords:** legged robot, locomotion control, frequency adaptation, motor pattern adaptation, central pattern generator

## Abstract

Existing adaptive locomotion control mechanisms for legged robots are usually aimed at one specific type of adaptation and rarely combined with others. Adaptive mechanisms thus stay at a conceptual level without their coupling effect with other mechanisms being investigated. However, we hypothesize that the combination of adaptation mechanisms can be exploited for enhanced and more efficient locomotion control as in biological systems. Therefore, in this work, we present a central pattern generator (CPG) based locomotion controller integrating both a frequency and motor pattern adaptation mechanisms. We use the state-of-the-art Dual Integral Learner for frequency adaptation, which can automatically and quickly adapt the CPG frequency, enabling the entire motor pattern or output signal of the CPG to be followed at a proper high frequency with low tracking error. Consequently, the legged robot can move with high energy efficiency and perform the generated locomotion with high precision. The versatile state-of-the-art CPG-RBF network is used as a motor pattern adaptation mechanism. Using this network, the motor patterns or joint trajectories can be adapted to fit the robot's morphology and perform sensorimotor integration enabling online motor pattern adaptation based on sensory feedback. The results show that the two adaptation mechanisms can be combined for adaptive locomotion control of a hexapod robot in a complex environment. Using the CPG-RBF network for motor pattern adaptation, the hexapod learned basic straight forward walking, steering, and step climbing. In general, the frequency and motor pattern mechanisms complement each other well and their combination can be seen as an essential step toward further studies on adaptive locomotion control.

## 1. Introduction

Adaptation is an essential aspect of legged locomotion. By skillfully manipulating the movement, animals can adapt their behavior in accordance with the environment, morphological variations, and external and intrinsic perturbations. This is a vital trait when moving in complex and unstructured environments where legs are especially advantageous. Within the robotics domain, there have been many attempts to create adaptive locomotion controllers. One promising approach is to use artificial central pattern generators (CPGs), inspired by their biological counterparts. These CPGs can generate rhythmic motor signals and are, when coupled with sensory feedback, also able to adapt their outputs accordingly.

Frequency adaptation is one branch of CPG-based adaptation techniques that have also been observed in animals such as cockroaches (Bender et al., 2011), stick insects (Cruse, 1990; Grabowska et al., 2012), and cats (Forssberg and Grillner, 1973). Frequency adaptation mechanisms seek to optimize the movement speed of robots based on feedback (entrainment). The consequences of using a wrongly tuned CPG frequency include loss of precision, unwanted movement, and energy-inefficient locomotion (Thor and Manoonpong, 2019a). Frequency adaptation mechanisms are often realized by expanding CPGs with local sensory feedback for robot locomotion (Aoi et al., 2011; Fukuoka et al., 2015; Owaki and Ishiguro, 2017; Ambe et al., 2018). Moreover, as frequency and phase are related, many works on frequency adaptation also inherit the adaptation of different walking gaits or interlimb coordination (for a detailed review, see Aoi et al., 2017). In this work, the frequency and phase are separated and do not affect each other.

Berendes et al. (2016) measured the coupling between the amputated limbs of a Drosophila and intact legs, allowing them to highlight the role of sensory feedback across walking speeds. In their experiments, low frequency walking showed almost no cycle-to-cycle coupling between stumps and intact legs as compared to the strong coupling displayed during high frequency walking. The finding that sensory feedback is more important for slow walking animals is reinforced by Mantziaris et al. (2017) in their study on deafferented stick insects. They found that while the CPG networks interact on a pre-motor level to create coordination patterns, these outputs are not observed in live animals, leading to their conclusion that sensory feedback is required to shape the basic coordination pattern produced by the neural architecture. On the contrary, Sponberg and Full (2008) establish that sensory feedback is less important for the faster walking cockroach. They observed the gait of a cockroach running on rough vs. smooth terrain. Even though the speed, pitch, roll, and yaw of the body varied when running on rough terrain, the animal maintained an alternating tripod gait, indicating that sensory feedback was not contributing in a significant way. Our study finds that frequency also slows down during complex motion when sensory feedback is more necessary.

Another important branch is motor pattern adaptation, where the goal is to find motor patterns or joint trajectories that solve a specific task or enable the robot to move in a certain way. First of all, it is essential for legged robots that the motor pattern is adapted to their morphology since different robots require different motor patterns. Moreover, suppose the robot is to be used in non-ideal environments. In that case, it is also necessary that the motor pattern can be adapted online to deal with the environment and external perturbations. Furthermore, it could be a requirement to learn new motor patterns for new tasks. Motor pattern adaptation has been applied both to CPG-based locomotion controllers (Nakanishi et al., 2004; Oliveira et al., 2011) and deep neural network locomotion controllers (Clune et al., 2011; Hwangbo et al., 2019; Lee et al., 2020; Schilling et al., 2020a,b; Yang et al., 2020).

Although the techniques mentioned above have been investigated and applied to robot locomotion control, they are rarely combined (Aoi et al., 2017). Thus, their work stays at a conceptual level where a single adaptation is shown without putting it into a bigger picture and seeing the coupling effect between methods. However, we hypothesize that if the adaptation mechanisms can be combined and appropriately exploited, the locomotion behaviors of legged robots will become enhanced and more efficient like their biological counterparts. To address this hypothesis, we propose for the first time an adaptive CPG-based locomotion controller that combines two state-of-the-art CPG adaptation mechanisms for frequency and motor pattern adaptations. While the two fundamental adaptation mechanisms have been proposed and implemented on artificial legged systems, they are employed separately. Their synergy or coupling has not been fully addressed and validated on a legged system because mutual adaptation can lead to conflict, involve complex dynamical processes, and possibly require different adaptation time scales.

**Figure 1:**
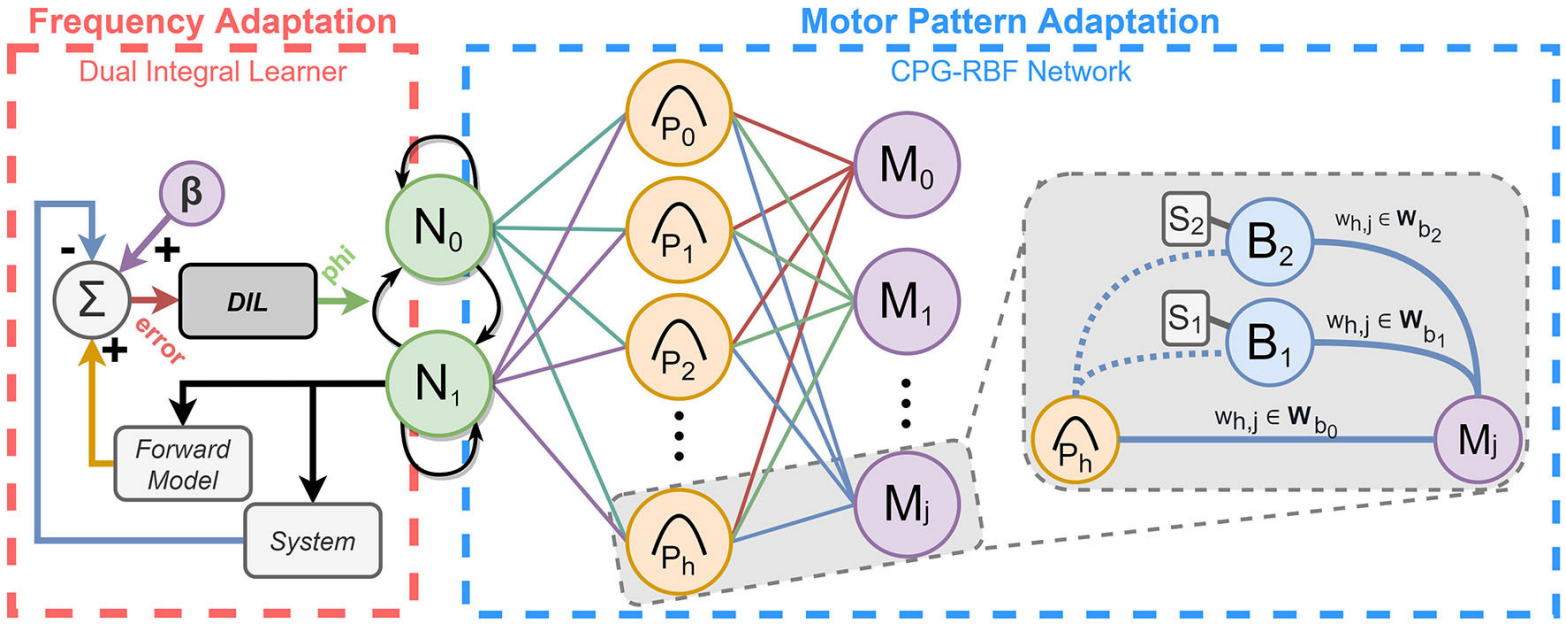
Combining frequency adaptation (DIL) and motor pattern adaptation (CPG-RBF network) into an integrated adaptive CPG-based locomotion controller.

As a frequency adaptation mechanism, we use the error-based Dual Integral Learner (DIL) presented in Thor and Manoonpong (2019a,b). In this approach, the tracking errors between desired and actual positions of the joints are used to adapt a CPG frequency such that the legged robot's locomotion speed matches its motor performance (see Figure 1). The consequences of running the system at too high a frequency (i.e., high tracking error) are, as mentioned previously, loss of precision, unwanted movement, energy inefficiency, and in the worst-case, motor collapse (Thor and Manoonpong, 2019b). Thus, low tracking error is especially important for motor pattern adaptation where the generated trajectories are excreted to be followed. In Thor and Manoonpong (2019a,b), the error-based DIL approach has shown good performance on various robots where it can quickly adapt the CPG frequency for energy-efficient locomotion with low tracking error, prevent leg damage and deal with a variety of motor performances.

As a motor pattern adaptation mechanism, we use the modular CPG-RBF network proposed in Thor et al. (2020) and Thor and Manoonpong (2021). The CPG-RBF network comprises a CPG and radial basis function (RBF) network (see Figure 1). Combining the CPG-RBF network with the DIL mechanism for frequency adaptation is, therefore, straightforward. The main features of the CPG-RBF network are as follows: it is simple, minimal, and intuitive to use; it has few control parameters resulting in fast learning; it is scalable; finally, it is generic and thus can be applied to legged robots with different morphologies (Thor et al., 2020). Furthermore, the network is modular, and new sub-behaviors or sensorimotor coordination can be learned independently and added on the fly. In this work, the robot learns three different motor patterns using an open-loop behavior for walking straight, a closed-loop sub-behavior module for steering, and a closed-loop sub-behavior module for climbing steps. It is important to note that while (Thor and Manoonpong, 2021) investigate the possibility of creating a modular CPG-based mechanism with motor pattern adaptation, we investigate the possibility and advantage of combining such a mechanism with an additional CPG-based adaptation mechanism for frequency adaptation.

In the following sections, we provide a detailed introduction to both the DIL mechanism for frequency adaptation (section 2.1) and the CPG-RBF network for motor pattern adaptation (section 2.2). We then present the novel locomotion controller, comprising both the frequency and motor pattern adaptation mechanism (section 2.3). Finally, we evaluate the proposed locomotion controller on the Modular Robot Framework (MORF) (Thor et al., 2018) in a complex environment (sections 3 and 4). MORF is a 0.42 m long hexapod robot weighing 4.2 kg. Each leg is 0.25 m long when fully stretched and has three actuated degrees of freedom, resulting in a total of 18 degrees of freedom. Each degree of freedom is controlled with a Dynamixel XM430 coreless electric motor set to position mode using the built-in PID controller.

## 2. Materials and Methods

### 2.1. CPG Network With Frequency Adaptation

The core of both the frequency and motor adaptation mechanisms is an artificial CPG. As mentioned in the introduction, CPGs can generate rhythmic motor signals but may also use sensory feedback to adapt their outputs. A biological CPG is a group of nerve cells or interconnected neurons within the thoracic ganglia of invertebrates and spinal cords of vertebrates (Biewener, 2003). These CPGs are used for locomotion and other rhythmic movements such as chewing, swallowing, or breathing (Aoi et al., 2017; Nachstedt et al., 2017; Grillner and El Manira, 2020). Over the years, various artificial CPG models with differing degree of complexity have been proposed: conceptual biological models (Brown, 1914), detailed biophysical models (Hellgren et al., 1992), connectionist models (Ekeberg, 1993), and abstract models (Ijspeert, 2008; Yu et al., 2014). Within the field of robot control, most studies have implemented abstract CPG models due to their simplicity and many advantages (Ijspeert et al., 2007; Manoonpong et al., 2013b; Spröwitz et al., 2013; Fan et al., 2016; Nordmoen et al., 2019; Degroote et al., 2020; Thor and Manoonpong, 2021). From a control perspective, the advantages of CPGs are their robustness against perturbations, easy and smooth frequency modulation, suitability for distributed implementation, and the fact that they use few control parameters.

Both the frequency and motor pattern adaptation mechanisms, presented in the following sections, use the abstract neural SO(2)-oscillator based CPG model (Pasemann et al., 2003). The SO(2)-based CPG network comprises two fully-connected standard additive discrete-time neurons (*N*_0_ and *N*_1_ in Figure 1) both using a sigmoid transfer function. The outputs of the two neurons in the SO(2)-oscillator are given by


(1) $$o_{i}(t+1)=\tanh\left(\sum_{j\;=\;0}^{N}w_{ij}(t)o_{j}(t)\right),$$

where *o*_*i*_ is the output from neuron *i*, *N* is the number of neurons, and *w*_*ij*_ is the synaptic weight from neuron *i* to *j*. The two neurons produce rhythmic outputs with a phase shift of π/2.

As proven in Pasemann et al. (2003), the CPG network produces a quasi-periodic output when the weights are selected as


(2) $$\left(\begin{array}{cc} w_{00}(t) & \;w_{01}(t)\\ w_{10}(t) & \;w_{11}(t) \end{array}\right)=\alpha \cdot \left(\begin{array}{cc} \cos\;\varphi (t) & \;\sin\varphi (t)\\ -\sin\;\varphi (t) & \;\cos\varphi (t) \end{array}\right),$$

with 0 < φ(*t*) < π as the frequency-determining parameter. Parameter α determines the amplitude and the non-linearity of the output oscillations.

The frequency-determining parameter of the SO(2)-oscillator (φ) can be easily and smoothly modulated online. To adapt φ for energy-efficient and accurate locomotion we use the error-based DIL approach proposed in Thor and Manoonpong (2019a,b) (see frequency adaptation in Figure 1). The DIL mechanism comprises slow and fast learners, acting on different time-scales in parallel. Both the fast and slow learners receive the same error and incorporate a proportion of it into their current estimation of the perturbation (Wolpert et al., 2011). This is shown in the following equations:


(3) $$\begin{array}{l} x_{f}(n)=A_{f}\cdot x_{f}(n-1)+B_{f}\cdot e(n)+C_{f}\cdot \int e(n)\\ \;x_{s}(n)=A_{s}\cdot x_{s}(n-1)+B_{s}\cdot e(n)+C_{s}\cdot \int e(n)\\ \;e(n)=(f(n-\Delta S)-s(n))+\beta \\ \;x(n)=x_{s}(n)+x_{f}(n)\\ \;\varphi (n)=\varphi_{0}-x(n) \end{array}$$

where *e*(*n*) is the joint tracking error calculated as the difference between the forward model (*f*(*n* + Δ*S*)) with the system delay (Δ*S*) and system output (*s*(*n*)). The forward model is used to translate the CPG output and sensory feedback into expected joint positions. Since the CPG-RBF network does exactly this, it can itself be used as the forward model. The output of the forward model or CPG-RBF network is compared to the system output, which is the joint position feedback from the actual joints on the robot. The system delay (Δ*S*) is used to account for the delay between sending the joint position command to it being executed on the robot. In this way, the forward model output (i.e., desired joint position) and system output (i.e., actual joint position) can be directly compared. It should be noted that a small bias (β = 0.02) is added to the error. In this way, the DIL mechanism will receive a positive tracking error when the forward model and system joint positions match and try to increase the CPG frequency. In other words, the β-parameter also enables the DIL mechanism to increase the CPG frequency when the system can run at a faster frequency without increasing the tracking error. *x*_*f*_(*n*) and *x*_*s*_(*n*) are the states of the fast and slow learners, each consisting of three terms with *x*(*n*) being the learner output. The computation of each learner state is straightforward. The first term is the product of the previous learner state (*x*_*f,s*_(*n* − 1)) and a constant retention factor (*A*_*f,s*_). The second term is the product of the error feedback (*e*(*n*)) and a constant learning rate (*B*_*f,s*_). The final term is a product of the integrated or summed error (∫*e*(*n*)) over time and a constant integral rate (*C*_*f,s*_). The parameters *A*_*f,s*_, *B*_*f,s*_, and *C*_*f,s*_ are under the constraints of *A*_*f*_ < *A*_*s*_, *B*_*f*_ > *B*_*s*_, and *C*_*f*_ > *C*_*s*_. Thus the fast learner learns more rapidly as indicated by a higher learning rate but also forgets more rapidly as indicated by a lower retention factor. The main advantages of the dual learner setup include fast and stable learning, savings in relearning, tracking error reduction, and spontaneous recovery of previously learned parameters (Thor and Manoonpong, 2019a). In this work, we use *A*_*f*_ = 0.35, *A*_*s*_ = 0.7, *B*_*f*_ = 0.2, *B*_*s*_ = 0.02, *C*_*f*_ = 0.001, and *C*_*s*_ = 0.0001.

When used for frequency adaptation, the DIL mechanism calculates a new CPG frequency (φ(*n*)) as the difference between the dual learner output (*x*(*n*)) and an arbitrary initial CPG frequency (φ_0_). In this way, the CPG frequency will be adapted to minimize the joint tracking error (*e*(*n*)). When the tracking error is high, the CPG frequency decreases, such that the joints are given more time to track the desired target positions. In Thor and Manoonpong (2019a,b), the tracking error is calculated by comparing the desired and actual joint position amplitudes using post-processing units. Recall, that the actual joint positions are given by the system output, while the desired joint positions are calculated using a forward model that can translate the CPG outputs into expected sensor signals. In this work, the process of calculating the tracking error is greatly simplified. By implementing the system delay (Δ*S*) into the forward model (*f*(*n*)), the actual and desired joint positions can be compared directly, thus removing the need for post-processing units to extract the amplitude. This also enables the frequency adaptation mechanism to work with motor pattern adaptation mechanisms, where the amplitude of the arbitrarily shaped motor patterns can be difficult to calculate online. As input to the DIL mechanism, the low-pass filtered summation of the absolute tracking error for all joints is used.

### 2.2. CPG Network With Motor Pattern Adaptation

In this work, the CPG-RBF network is used for motor pattern adaptation (see motor pattern adaptation in Figure 1). The CPG-RBF network consists of a SO(2)-based CPG and an RBF network to reshape the otherwise fixed wave-shaped CPG output. The RBF network is a three-layered neural network that uses radial basis activation functions (Broomhead and Lowe, 1988). In the case of the CPG-RBF network, two-dimensional Gaussian functions are used with transfer functions that can be described as


(4) $$o_{P_{h}}=e^{-\left(\frac{{\left(o_{0}-\mu_{h,0}\right)}^{2}+{\left(o_{1}-\mu_{h,1}\right)}^{2}}{\sigma_{RBF}^{2}}\right)},$$

where, μ_*h*, 0_ and μ_*h*, 1_ are two means of RBF neuron *P*_*h*_, $\sigma_{RBF}^{2}$ is the common variance, and *o*_*P*_*h*__ is the output of the RBF neuron when receiving inputs *o*_0_ and *o*_1_ from the CPG. The two means are manually chosen such that the RBF kernels are equally distributed along one CPG signal period (Thor et al., 2020). This is achieved by


(5) $$\mu_{h,n}=o_{n}\left(\frac{(h-1)\cdot T}{H-1}\right),$$

where *n* is the CPG output index, *T* is the period of the CPG signal (*T* ≈ 1/0.30 *Hz*), and *H* is the total number of RBF neurons. The advantages of equally distributing the means along one period of the CPG output signal are that it is possible to modify discrete parts of the CPG signal shape and the means of the neurons do not need to be learned (Thor et al., 2020).

The reshaping of the CPG signal is encoded in the weights of the synapses connecting the RBF layer to the motor neurons (*W*_*b*_0__ in Figure 1). The complexity of the output signal is controlled by the number of RBF neurons, *H*. A high number of neurons enables complex output signals, while a small number can only produce simple signals. However, the use of many RBF neurons also results in slower convergence when learning the synaptic weights. While the number of RBF neurons controls the signal complexity, their variance, $\sigma_{RBF}^{2}$, controls its smoothness. A low variance results in high frequency outputs, while a higher one results in smooth signals. This creates a trade-off, and as in Thor et al. (2020), we use *H* = 20 and $\sigma_{RBF}^{2}=0.04$, allowing the learning of smooth, complex control policies at acceptable convergence rates. Furthermore, the CPG-RBF network can automatically scale to different CPG frequencies as the RBF neuron's activation encodes the joint position at a particular phase in the stepping cycle. This means that the motor pattern will keep the same shape for different frequencies. This is also what allows it to be combined with the frequency adaptation mechanism.

The weight set *W*_*b*_0__ encodes an open-loop behavior for walking straight. To also facilitate online motor pattern adaptation, sub-behavior modules (see gray box with neurons *B*_1−2_ in Figure 1) integrating sensory feedback (*S*_1−2_) are added in parallel to the synapses, connecting the RBF layer and motor neurons (Thor and Manoonpong, 2021). The parallel neurons are shunting inhibition neurons, set up such that their outputs are the product of the RBF neuron outputs and sensory feedback. Based on the sensory feedback and weights of the synapses connecting the parallel neurons to the motor neurons (*W*_*b*_1__ and *W*_*b*_2__ in Figure 1), these parallel modules can modify the already learned open-loop motor pattern output. With the parallel closed-loop sub-behavior modules, the motor neuron output can be formulated as


(6) $$M_{j}=o_{P_{h}}\cdot W_{b_{0}}+\left(\sum_{n\;=\;1}^{2}o_{P_{h}}\cdot S_{n}\cdot W_{b_{n}}\right),$$

where *M*_*j*_ is the motor pattern output to motor *j* and *o*_*P*_*h*__ is the output from RBF neuron *P*_*h*_.

Due to its flexibility, the CPG-RBF network can be implemented as a centralized or decentralized controller. When implemented as a central controller, all joints in a leg will learn unique trajectories but they will be the same across all legs. In this case, the phase relationship between legs needs to be predefined. When the controller is decentralized, the individual legs will learn different joint trajectories and increasingly complex control policies can be learned. However, decentralization comes at the price of additional policy parameters, and thus a slower convergence (for details, see Thor et al., 2020). In this work, we implement the CPG-RBF network as a centralized controller because of its simplicity and use a fixed leg phase relationship where contralateral legs operate in a reciprocal fashion, resulting in a tripod gait. This gait behavior is also often seen in walking animals (Biewener, 2003).

To learn the weights of the CPG-RBF network (*W*_*b*_0__, *W*_*b*_1__, and *W*_*b*_2__), the state-of-the-art simplistic learning mechanism PI^BB^ (Stulp and Sigaud, 2013) is employed. The PI^BB^ is a probability-based black box optimization approach that follows a direct policy search to improve the policy parameters with respect to a reward function. The reward function rewards intended behaviors (e.g., walking fast) and penalizes unwanted ones (e.g., unstable movement) such that the behavior or policy is trained to exhibit the intended behaviors. In this work, we use three different task specific reward functions, as explained in section 3. The complete explanation and pseudocode for PI^BB^ and how the reward functions are used can be found in the Supplementary Material.

### 2.3. CPG Network With Frequency and Motor Pattern Adaptation

Combining the frequency and motor pattern adaptations is straightforward (see Figure 1). The simplicity of combining the adaptation mechanisms is not only because both use an artificial CPG but also because the DIL can work on arbitrarily shaped signals, due to the modification introduced in this work, and the CPG-RBF network automatically scales with respect to frequency.

The overall adaptation process can be described as follows. First, the weights of the CPG-RBF network and its sub-behavior modules are learned using a low CPG frequency. This ensures that motor patterns can be learned with high precision and without tracking error. Next, the DIL is added to the CPG in order to adapt the CPG frequency online. The DIL will increase and decrease the frequency based on the motor pattern, environment, and external or internal perturbations to keep the tracking error low and the frequency at the highest possible level. This frequency can be seen as the resonance frequency because it is the highest frequency at which the system moves with the highest possible amplitude or lowest tracking error.

### 2.4. Simulation Environment

For evaluating the locomotion controller on MORF, we use the robot simulation framework CoppeliaSim from Coppelia Robotics (Rohmer et al., 2013) with the Vortex physics engine by CM Labs. CoppeliaSim offers real-world parameters (i.e., corresponding to physical units) for many physical properties, making it both realistic and precise. The MORF robot is set up in simulation such that its morphology, weight, sensors, and motor performance match that of the real robot. Both the control loop frequency and simulation step size are set to 60 Hz. Finally, all communication with the controller and simulation is implemented in the robot operating system (ROS) such that the controller can be easily transferred to a physical version of MORF in future work.

## 3. Results

### 3.1. Motor Pattern Adaptation

**Figure 2:**
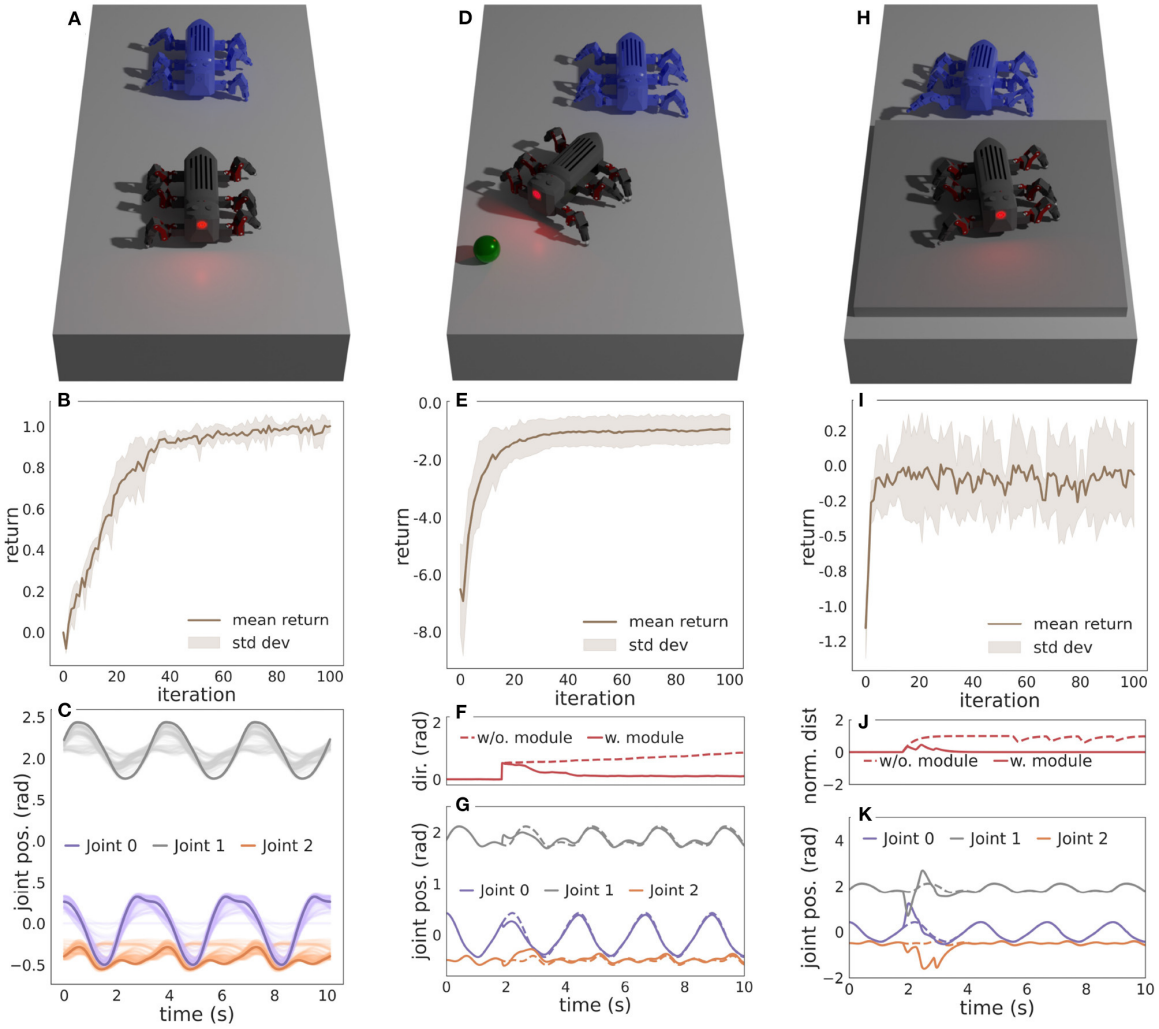
**(A)** The simulated environment and learned open-loop behavior for walking straight. **(B)** The mean reward and standard deviation per iteration. **(C)** The motor patterns during learning for a single leg. The solid lines are converged patterns, and the transparent lines are intermediate patterns during learning. **(D)** The simulated environment and learned closed-loop sub-behavior module for steering. A sphere specifying the desired heading direction will spawn after 2 s. **(E)** The mean reward and standard deviation per iteration. **(F)** The heading direction error with and without the sub-behavior module. **(G)** The learned motor patterns for a single leg with and without the sub-behavior module enabled. **(H)** The simulated environment and learned closed-loop sub-behavior module for climbing steps. **(I)** The mean reward and standard deviation per iteration. **(J)** The normalized optic distance sensor feedback with and without the sub-behavior module enabled. **(K)** The learned motor patterns for a single leg with and without the sub-behavior module enabled. For **(A,D,H)** the blue MORF shows an earlier time-step of the sub-behavior. Modified from Thor and Manoonpong (2021).

To assess the performance of the proposed locomotion controller with frequency and motor pattern adaptation, we start by learning three motor pattern behaviors with the CPG-RBF network: an open-loop behavior and two closed-loop behaviors. The open-loop behavior (*W*_*b*_0__) can be considered as the base behavior as it lays the foundation for the two closed-loop behaviors. The open-loop behavior encodes a basic straight forward walking behavior without requiring any sensory feedback, and it is learned in a simple environment without any obstacles, as shown in Figure 2A. After learning this behavior, the two closed-loop sub-behavior modules can be learned in parallel with the open-loop behavior. The first closed-loop sub-behavior module encodes a steering behavior (*W*_*b*_1__). It uses heading orientation sensory feedback from an inertial measurement unit and compares it to the desired heading direction to get an error (*S*_1_). A negative heading error is projected to the legs on the right side and a positive to the legs on the left side. The sub-behavior is trained to minimize this error in an environment where a sphere specifying the desired heading direction will spawn after 2 s, as shown in Figure 2D. The second closed-loop sub-behavior module encodes a step climbing behavior (*W*_*b*_2__). It uses binary sensory feedback from an optic distance sensor mounted at the front of MORF. The sensor feedback is filtered using three low-pass single-pole infinite impulse response (IIR) filters in series. Putting the IIR filters in series enables memory and, consequently, the ability to retain the sensory feedback for some time. The sensory feedback is only projected onto the two front legs as they are responsible for lifting the body over the step. The step climbing behavior is trained in an environment where MORF walks toward a 0.04m thick plate, as shown in Figure 2H. The overall modular design and learning parameters are similar to those used in Thor et al. (2020) and Thor and Manoonpong (2021). This includes a fixed walking frequency of 0.30 Hz (φ ≈ 0.01π) during learning. All three motor patterns are learned five times for 100 iterations to calculate the mean and standard deviation of the return.

#### 3.1.1. Learning Open-Loop Behavior for Walking Straight

The open-loop behavior for walking straight is learned in the simulated environment shown in Figure 2A using the following reward function:


(7) $$R_{k}=w_{d}\cdot d-(w_{\gamma }\cdot \gamma +w_{\xi }\cdot \xi +w_{\varsigma }\cdot \varsigma ).$$

where *w*_*d*_ = 3, *w*_γ_ = 1, *w*_ξ_ = 3, and *w*_ς_ = 0.75. The reward function consists of several measures: distance (*d*), instability (γ), body height error (ξ), and slippage (ς). The distance measure rewards fast straight locomotion, while the instability measure penalizes instability during movement. It comprises the sum of variance in body tilt, roll, pan, and body height. A pan of 0° means that MORF is walking straight ahead, whereas a tilt and roll equal to 0° means that MORF is parallel with the ground. Instability thereby penalizes movements that are not in the walking direction. The body height error is measured as the difference between mean body height during walking and the desired walking height. The slippage sub-reward considers how much each leg of the robot slips on the ground. The slippage return is unitless and calculated as the amount a leg tip moves (i.e., having a velocity greater than some threshold) while in ground contact (i.e., when the leg tip and walking surface are in collision). The slippage is normalized between 0 and 1 and the leg with the highest slippage is used as return. A slippage return of 1 thus implies that one or more legs slip on the ground whenever in contact with it. Note that each measurement is multiplied by a weight (*w*_*d*_, *w*_γ_, *w*_ξ_, and *w*_ς_) to ensure similarity in magnitude and range. The instability measure is limited at 8 to avoid negative returns becoming too large. In Equation (7), the distance measurement can be regarded as the dominating reward. Finally, the open-loop behavior is learned using an exploration noise (ϵ_*k*_) of 0.02 and a roll-out execution time of 6 s.

Figure 2B shows the mean reward and standard deviation for each iteration, and Figure 2C shows the resulting motor patterns during learning.

#### 3.1.2. Learning Closed-Loop Sub-behavior Module for Steering

The closed-loop sub-behavior module for the steering behavior is learned in the simulated environment shown in Figure 2D using the following rewards function:


(8) $$R_{k}=w_{d}\cdot d-(w_{\gamma }\cdot \gamma +w_{\xi }\cdot \xi +w_{\varsigma }\cdot \varsigma +w_{\delta }\cdot \delta ).$$

where *w*_*d*_ = 0.1, *w*_γ_ = 1, *w*_ξ_ = 3, *w*_ς_ = 1 and *w*_δ_ = 6. The reward function is similar to that of the open-loop behavior for walking straight but uses a lower *w*_*d*_ and higher *w*_ς_. Moreover, a heading direction error (δ) that measures the difference between target and current heading direction is included to penalize movement not in the target direction. Finally, the sub-behavior module for steering is learned using an exploration noise (ϵ_*k*_) of 0.02 and a roll-out execution time of 10 seconds.

Figure 2E shows the mean reward and standard deviation for each iteration, while Figure 2F shows the heading direction error with and without the learned sub-behavior module. Finally, Figure 2G shows the motor patterns with and without the learned sub-behavior module. Here, the motor patterns of the open-loop behavior are modulated to steer the robot toward the target.

#### 3.1.3. Learning Closed-Loop Sub-behavior Module for Climbing Steps

The closed-loop sub-behavior module the step climbing behavior is learned in the simulated environment shown in Figure 2H using the following rewards function:


(9) $$R_{k}=w_{d}\cdot d-(w_{\gamma }\cdot \gamma +w_{\xi }\cdot \xi +w_{\varsigma }\cdot \varsigma ).$$

where *w*_*d*_ = 0.5, *w*_γ_ = 1, *w*_ξ_ = 0, and *w*_ς_ = 0.5. Again, the reward function is similar to the open-loop behavior for walking straight, but this time with the exclusion of the body height error measure (ξ), a lower *w*_*d*_, and a lower ς. The reason for not using the body height error measure is that it will penalize MORF when crawling onto a step. Finally, the closed-loop sub-behavior module for climbing steps is learned using an exploration noise (ϵ_*k*_) of 0.02 and a roll-out execution time of 14 s.

Figure 2I shows the mean reward and standard deviation for each iteration, while Figure 2J shows the normalized distance measured by the optic distance sensor with and without the learned sub-behavior module. Figure 2K shows the motor patterns with and without the learned sub-behavior module. Here, the motor patterns of the open-loop behavior are modulated to step onto the platform.

### 3.2. Online CPG Frequency Adaptation

**Figure 3:**
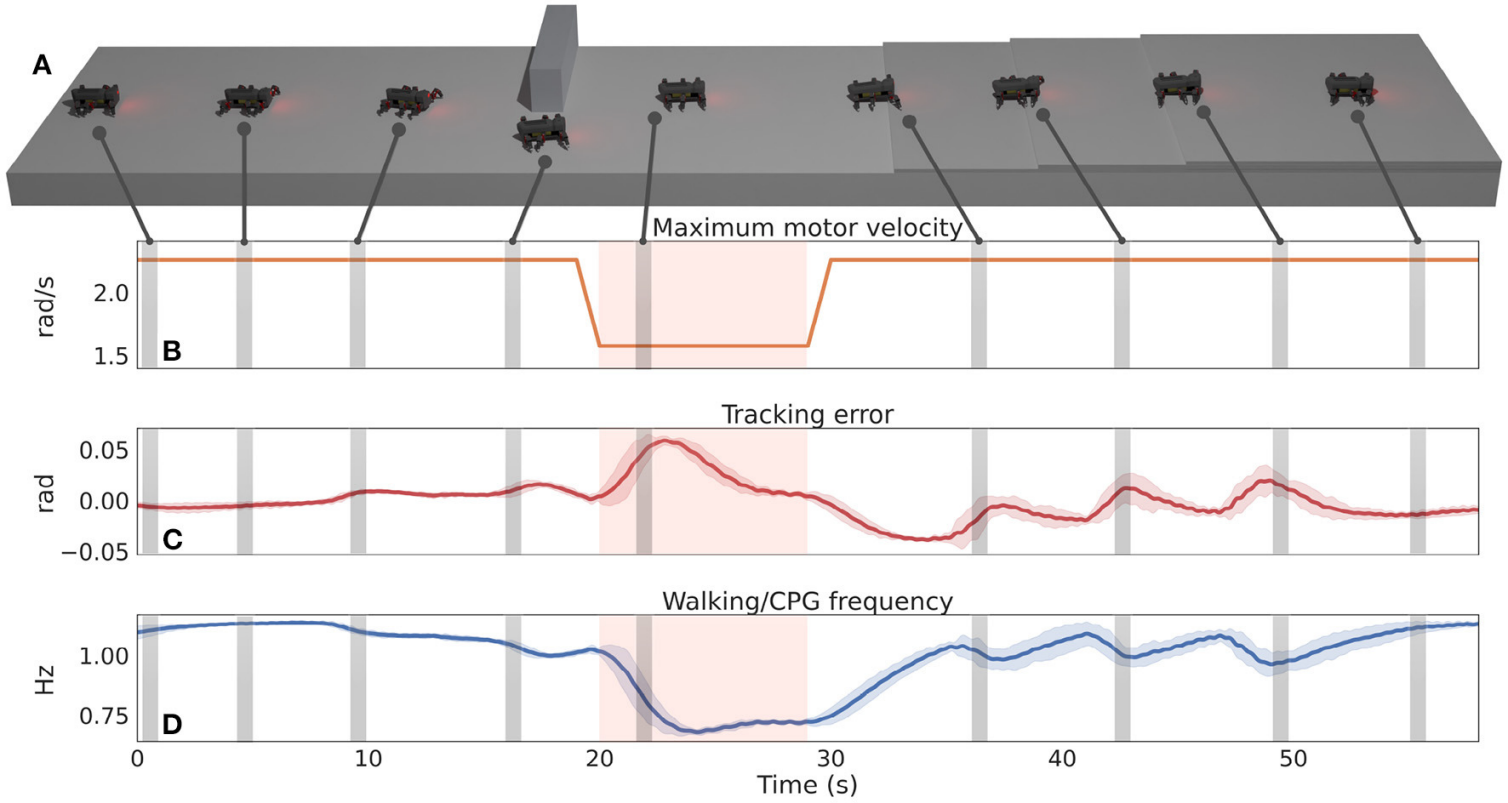
**(A)** The simulated environment and nine snapshots of MORF when using the locomotion controller with frequency and motor pattern adaptation. Each snapshot is highlighted on the plots below. **(B)** The motor performance (i.e., maximum velocity), which is lowered by 30% when MORF has passed the wall until it reaches the first step (red zone on the plots). **(C)** The mean tracking error. **(D)** The CPG frequency.

After learning the CPG-RBF network weights, the DIL is added to the CPG for online frequency adaptation. The combined controller is tested repeatedly 10 times in the environment shown in Figure 3A where MORF will need to use all the learned motor patterns. The walking path of MORF is predetermined but could be provided by high-level control algorithms like the pathfinding algorithms in Patle et al. (2019) and Goldschmidt et al. (2017) for increased autonomy. When MORF passes the wall (snapshot 4 in Figure 3A), the motor performance or maximum motor velocity is reduced by 30% for 1.5 m, as shown in Figure 3B. Consequently, the walking frequency has to be adapted to minimize the tracking error.

Figure 3C shows the mean tracking with standard deviation for the 10 trials. It is low-pass filtered, and the β-parameters have been subtracted, enabling the DIL to increase the frequency as explained in section 2.1. Recall that the input to the DIL mechanism is the low-pass filtered summation of the absolute tracking error for all joints. Figure 3D shows the mean walking frequency with standard deviation. The DIL modulates the walking frequency online based on the environment, motor pattern, and motor performance to reduce the tracking error. Overall, the results show that the DIL quickly adapts the walking frequency to 1.13 Hz for straight walking (snapshot 1). In snapshots 3–5, the frequency is lowered to enable complex motor patterns for steering with low tracking error. In snapshot 5, the motor performance (i.e., maximum velocity) is lowered by 30%, and the frequency is adapted accordingly. Finally, in snapshots 6–9, the frequency is lowered several times to enable complex motor patterns for climbing steps with low tracking error.

### 3.3. Comparing to Fixed Frequencies

Two experiments using fixed frequencies are additionally conducted and repeated ten times to show the effect and advantage of using frequency adaptation. The first fixed frequency is set to 0.30 Hz, which can be considered a base case since it is equal to the one used when learning the control modules of the CPG-RBF network. 0.30 Hz is a relatively low frequency where the motor patterns can be followed with no error. The second fixed frequency is set to 1.6 Hz, which is used to test how a high walking frequency impacts the performance of the controller.

**Figure 4:**
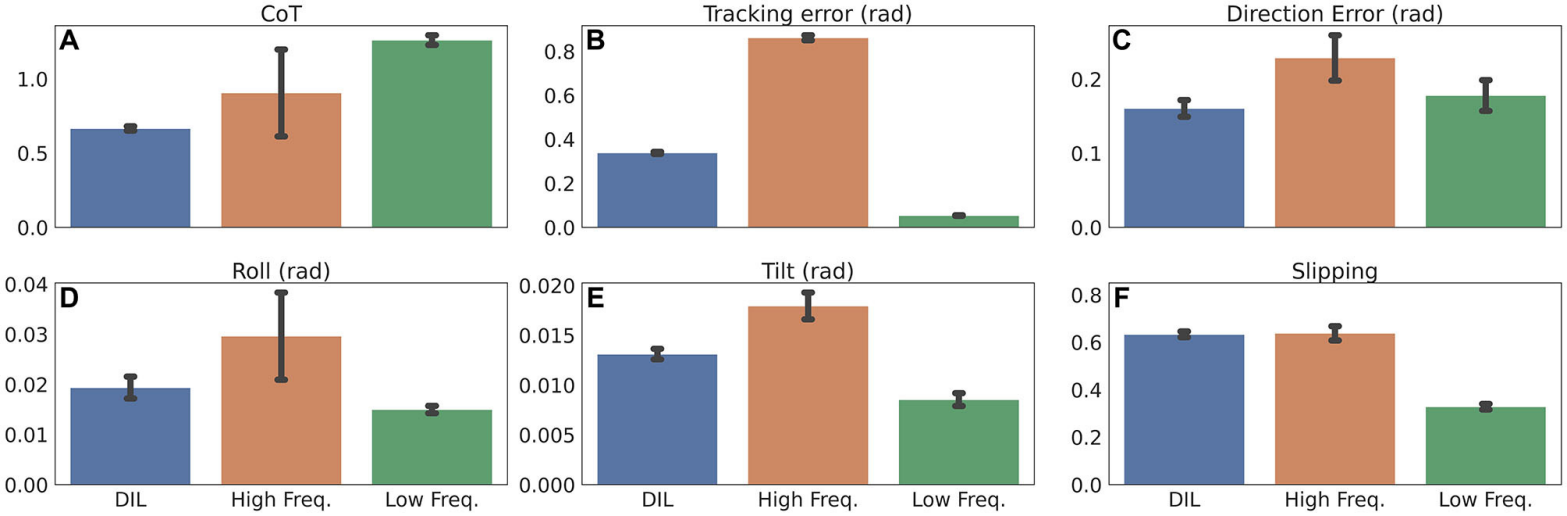
Performance measurement when using online CPG frequency adaptation (DIL), a fixed high frequency, and a fixed low frequency. **(A)** The mean CoT. **(B)** The mean raw tracking error. **(C)** The mean heading direction error. **(D)** The mean roll of MORF. **(E)** The mean tilt of MORF. **(F)** The mean slippage. All measurements are shown with standard deviation.

First, we compare the cost of transport (CoT). CoT is a dimensionless measurement that quantifies the energy efficiency of moving an animal, robot, or vehicle from one place to another. In this work, we calculate CoT as $\frac{P}{m\cdot g\cdot v}$, where *m* is the weight of the entire robot in *kg* (4.2 kg), *g* is the gravity of earth (9.82 *m*/*s*^2^), *v* is the walking velocity of the robot in *m*/*s*, and *P* is the power given as the joint torque in *N*·*m* times the angular joint velocity in *rad*/*s*. Figure 4A shows that when using frequency adaptation, the CoT is significantly lower than both a fixed low frequency (*p* > 0.999) and fixed high frequency (*p* = 0.983), with the fixed high frequency having the lowest CoT of the two (*p* = 0.998).

Next, we compare the tracking error. The tracking error presented in Figure 4B is raw, and does not include either the β-parameter or low-pass filtering as in Figure 3C. As expected, using a fixed low frequency does results in a lower tracking error than both frequency adaptation (*p* > 0.999) and a fixed high frequency (*p* > 0.999). Comparing frequency adaptation with a fixed high frequency shows that the former results in the lowest tracking error (*p* > 0.999).

Finally, we compare key performance measurements used when learning the weights of the CPG-RBF network. The first measurement, shown in Figure 4C, is the heading direction error used when learning the sub-behavior module for steering. Frequency adaptation resulted in a significantly smaller direction error than when using a fixed low frequency (*p* = 0.974) or a fixed high frequency (*p* > 0.999). The next measurements, shown in Figures 4D,E, are the roll and tilt of MORF, used to learn all three behaviors. The roll and tilt of MORF indicate how stable it is during walking. For both roll and tilt, a fixed low frequency resulted in the most stable locomotion when compared to frequency adaptation (*p*>0.999) and a fixed high frequency (*p* > 0.999). When comparing frequency adaptation and a fixed high frequency, it is clear that the former results in more stable locomotion for both roll (*p* = 0.997) and tilt (*p* > 0.999). The final measurement, shown in Figure 4F is slippage (the extent to which each leg of the robot slips on the ground), which is also used in the learning of all three behaviors. In this case, neither the use of frequency adaptation nor a fixed high frequency resulted in any significant difference (*p* = 0.266), while using a fixed low frequency resulted in less slippage (both with *p* > 0.999).

Besides the quantitative results, visual inspection shows that MORF has a hard time clearing the steps when using a fixed high frequency (see snapshots 6–8 in Figure 3). As shown in the Supplementary Video, its middle or hind legs became stuck for a few seconds. Out of 10 trials, each involving the climbing of three steps, MORF became stuck 66.67% of the time when using a fixed high frequency. In contrast, it never became stuck using frequency adaptation or a fixed low frequency. However, the use of low walking frequency leads to low energy efficiency (Figure 4A). Overall, the locomotion control with the motor pattern and online frequency adaptations offers the best compromise in comparison to motor pattern adaptation and a fixed frequency.

## 4. Discussion

In this work, we introduced a novel CPG-based locomotion controller, combining both motor pattern and frequency adaptations to enable a legged hexapod robot to efficiently navigate a complex environment. The frequency adaptation mechanism is error-based, meaning that it adapts the walking frequency such that the robot can follow the learned motor patterns with low error. The results show that when using the proposed controller, the frequency is adapted several times: initially for walking straight, then to variations in motor performance, and finally whenever closed-loop control modules enforce complex motor pattern adaptations. This is comparable to the biological behaviors observed in animal locomotion.

Compared to using fixed frequencies, frequency adaptation significantly reduces energy usage, as shown by the low CoT. This is because the frequency is adapted to the highest frequency at which the motor pattern is tracked with low error. Since this frequency is the highest at which the system has the highest amplitude (or lowest tracking error), it is comparable to the resonance frequency.

The results also show that tracking error plays a crucial role in executing the learned motor patterns, especially when adapted online, based on sensory feedback. This is demonstrated by the fact that when using a fixed high frequency where the tracking error is large, MORF could not consistently climb steps or achieve low direction error when turning. This was not a problem with a low tracking error, i.e., when using either a fixed low frequency or frequency adaptation. Furthermore, a low tracking error results in more stable locomotion (i.e., low roll and tilt movement) and a smaller direction error. Thus, the motor patterns emerging from the fact that the robot cannot follow the learned patterns are performing worse with regard to the performance measurements used as reward feedback during learning. As in Thor et al. (2020), where motor patterns are learned for a robot missing a leg, motor patterns could likewise be explicitly learned for the fixed high frequency. Although this would enable the robot to perform the behaviors more consistently, it would also make the motor pattern very frequency-dependent. This is because the motor patterns are learned to perform the desired behaviors with a specific tracking error. For a higher or lower tracking error, corresponding to a higher and lower CPG frequency, the performance of the desired behaviors cannot be guaranteed. For example, when lowering the frequency and removing the tracking error, the robot will use new and untested parts of the motor patterns. These parts are partly random as they were not included in the optimization process. In this work, we avoid frequency-dependent motor patterns and instead use the adaptive frequency mechanism to fit frequency to the learned motor patterns. Another reason for this approach is that while it takes a long time to learn the motor patterns, the frequency can be adapted online within seconds.

Although the energy efficiency, tracking error, stability, and direction error all improved when using frequency adaptation, the slippage measurement only improved when applying a fixed low frequency. There is no significant difference in slippage between frequency adaptation and a fixed high frequency. As can be seen from the results the tracking error is larger when using frequency adaptation compared to a fixed low frequency. As a consequence, frequency adaptation also results in higher tilt movement, roll movement, and slippage. However, the amount of tracking error when using frequency adaptation can be controlled by the β-parameter of the DIL mechanism. If the tracking error is vital to the system or task, the β-parameter could be tuned to accommodate this. However, tuning the β-parameter to obtain a low tracking error will be at the cost of slower convergence when increasing the frequency, and a trade-off thus exists.

Taken together, the results show that the frequency and motor pattern adaptation mechanisms can be combined for enhanced locomotion performance. When using a fixed low frequency (i.e., only motor pattern adaptation), the learned motor patterns can be followed with a low tracking error, which has many advantages but also low energy efficiency and high direction error. Using a fixed high frequency results in better energy efficiency when compared to a low frequency, but also a high tracking error resulting in behavior inconsistency and poor performance. When combining the motor adaptation mechanism with frequency adaptation, the frequency is adapted online, resulting in a good performance and high energy efficiency. Furthermore, the frequency adaptation mechanism is flexible since it can be tuned for either fast convergence or low tracking error.

For future studies, we plan to investigate parallel adaptation and learning. In Manoonpong et al. (2013a), correlation-based and reward-based learning was combined in neural control for policy improvement. The authors found that using the learning mechanisms in parallel compared to using a single learner resulted in higher performance. It would be interesting to see if it is likewise advantageous to use frequency adaptation in parallel with the learning of motor patterns. Finally, it would be interesting to investigate if any adaptation rule could be applied to the β-parameter of the DIL mechanism for increased performance.

## Data Availability Statement

The original contributions presented in the study are included in the article/Supplementary Material, further inquiries can be directed to the corresponding author/s.

## Author Contributions

MT and PM contributed to the conception and design of the work. MT developed the control mechanisms, performed the experiments, analyzed the data, and drafted the manuscript. PM helped with data analysis, reviewed the paper, supervised the whole project, and obtained the research funding. BS contributed to the manuscript in form of biological comparisons and reviewed the paper. All authors contributed to the article and approved the submitted version.

## Funding

This work was supported in part by the Human Frontier Science Program under grant agreement No. RGP0002/2017 (DLife) (PM, Project WP-PI) and in part by a startup grant on Bio-inspired Robotics from the Vidyasirimedhi Institute of Science and Technology (VISTEC) (PM, Project PI).

## Conflict of Interest

The authors declare that the research was conducted in the absence of any commercial or financial relationships that could be construed as a potential conflict of interest.

## Publisher's Note

All claims expressed in this article are solely those of the authors and do not necessarily represent those of their affiliated organizations, or those of the publisher, the editors and the reviewers. Any product that may be evaluated in this article, or claim that may be made by its manufacturer, is not guaranteed or endorsed by the publisher.
