# Supplementary material for: Locomotion Control With Frequency and Motor Pattern Adaptations
Source: Front Neural Circuits. 2021 Nov 25;15:743888. doi: 10.3389/fncir.2021.743888 (PMC8655109; doi:10.3389/fncir.2021.743888)
Supplement: Supplementary file 1 [file Data_Sheet_1.PDF]

Supplementary Material

1 PI^{BB} PSEUDOCODE

Algorithm S1 shows the pseudocode of PI^{BB}. The PI^{BB} algorithm executes K roll-outs, all with different Gaussian exploration noise, ϵ_k, added to the control policy parameters, $W_{b_j,k}$. The results from the K roll-outs are K returns, R_k, describing how well the policy, with added exploration noise, performed according to a certain reward function. Finally, the probability for each roll-out is calculated and used in cost-weighted averaging to update the policy parameters. The exploration noise is task-specific and the number of roll-outs (K) is set to 8 for all tasks. Both the exploration noise and the number of roll-outs are empirically chosen to promote fast and stable learning. The exploration noise is also linearly decayed during learning using a decay constant of $\gamma = 0.995$ for all tasks. Decaying the exploration noise allows for large initial weight changes and enforces smaller changes or fine-tuning toward the end of the learning process.

Algorithm S1 PI^{BB} Pseudocode

while *reward not converged* **do**
  // Execute K roll-outs
  for each $k \in K$ **do**
    // Sample in parameter space
    $\epsilon_k = \mathcal{N}(0, \sigma^2_{PI^{BB}})$
    // Execute policy and record final return (R)
    $R_k = \texttt{execCPGRBFN}(W_{b_j,k} + \epsilon_k)$
  end for
  // Calculate probability for each roll-out
  for each $k \in K$ **do**
    $S_k = e^{-\lambda \cdot \frac{R_k - \min_k(R_k)}{\max_k(R_k) - \min_k(R_k)}}$
  end for
  for each $k \in K$ **do**
    $P_k = \frac{S_k}{\sum_{k=1}^{K} S_k}$
  end for
  // Reward-weighted averaging
  $\delta W_{b_j,k} = \sum_{k=1}^{K} (P_k \cdot \epsilon_k)$
  // Update policy parameters
  $W_{b_j,k} \leftarrow W_{b_j,k} + \delta W_{b_j,k}$
  // Decay exploration noise
  $\sigma^2_{PI^{BB}} = \gamma \cdot \sigma^2_{PI^{BB}}$
end while
